# The effects of porosity on optical properties of semiconductor chalcogenide films obtained by the chemical bath deposition

**DOI:** 10.1186/1556-276X-7-483

**Published:** 2012-08-29

**Authors:** Yuri V Vorobiev, Paul P Horley, Jorge Hernández-Borja, Hilda E Esparza-Ponce, Rafael Ramírez-Bon, Pavel Vorobiev, Claudia Pérez, Jesús González-Hernández

**Affiliations:** 1 CINVESTAV-IPN Unidad Querétaro, Libramiento Norponiente 2000, Fracc. Real de Juriquilla, Querétaro, Qro, CP 76230, México; 2 CIMAV Chihuahua/Monterrey, Avenida Miguel de Cervantes 120, Chihuahua, Chih, CP 31109, México

**Keywords:** polycrystalline films, chalcogenide materials, nanopores, quantum confinement in pores

## Abstract

This paper is dedicated to study the thin polycrystalline films of semiconductor chalcogenide materials (CdS, CdSe, and PbS) obtained by ammonia-free chemical bath deposition. The obtained material is of polycrystalline nature with crystallite of a size that, from a general point of view, should not result in any noticeable quantum confinement. Nevertheless, we were able to observe blueshift of the fundamental absorption edge and reduced refractive index in comparison with the corresponding bulk materials. Both effects are attributed to the material porosity which is a typical feature of chemical bath deposition technique. The blueshift is caused by quantum confinement in pores, whereas the refractive index variation is the evident result of the density reduction. Quantum mechanical description of the nanopores in semiconductor is given based on the application of even mirror boundary conditions for the solution of the Schrödinger equation; the results of calculations give a reasonable explanation of the experimental data.

## Background

Chemical bath deposition (CBD) is a cheap and energy-efficient method commonly used for the preparation of semiconductor films for sensors, photodetectors, and solar cells. It was one of the traditional methods to obtain chalcogenide semiconductors including CdS and CdSe [1-6]. However, large-scale CBD deposition of CdS films raises considerable environmental concerns due to utilization of highly volatile and toxic ammonia. On the other hand, the volatility of ammonia modifies pH of the reacting solution during the deposition process, causing irreproducibility of thin film properties for the material obtained in different batches [1,3].

We manufacture CdS, CdSe, and PbS films using the CBD process to minimize the production cost and energy consumption. Ammonia-free CBD process was used to avoid negative environmental impact (see [7] reporting an example of CBD-made solar cell with structure glass/ITO/CdS/PbS/conductive graphite with quantum efficiency of 29% and energy efficiency of 1.6% ). All these materials have the melting temperatures above 1,000°C, remaining stable during the deposition process. It is also known that PbS is very promising for solar cell applications, confirmed by the recent discovery of multiple exciton generation in their nanocrystals [8].

Chemical bath-deposited films [9] have a particular structure. As a rule, at initial deposition stages, small (3 to 5 nm) nanocrystals are formed. They exhibit strong quantum confinement leading to large blueshift of the fundamental absorption edge. Historically, blueshift was first discovered namely in CBD-made CdSe films [9,10]. At later stages, the crystallite size becomes larger so that the corresponding blueshift decreases. Another feature characteristic to the process is a considerable porosity [3,9] inherent to the growth mechanism, which takes place ion by ion or cluster by cluster depending on the conditions or solution used (see also [11,12]). The degree of porosity decreases for larger deposition time because the film becomes denser. At the initial stage, the porosity can be up to 70% [9], and at final stages, it will be only about 5% to 10% .

In this paper, we present the experimental results for the investigation of porosity effects for relatively large deposition times upon the optical characteristics of CBD-made semiconductor materials such as CdS, CdSe, and PbS. We show that the nanoporosity can blueshift the absorption edge, leading to the variation observed for material with pronounced nanocrystallinity. For theoretical study of nanopores in a semiconductor, we use mirror boundary conditions to solve the Schrödinger equation, which were successfully applied to nanostructures of different geometries [13-15]. We show that the same treatment of pores allows to achieve a good correlation between theorical and experimental data.

## Methods

The authors successfully developed ammonia-free CBD technology for polycrystalline CdS, CdSe, and PbS films, described in detail elsewhere [4-7,11,12]. We characterize the obtained structures by composition, microstructure (including average grain size), and morphology using X-ray diffraction, SEM, and EDS measurements. Optical properties were investigated with UV–vis and FTIR spectrometers. All experimental methods are described in the aforementioned references, together with the detailed results of this complex material study. Here, we would like to discuss optical phenomena characteristic to the entire group of semiconductor film studied, skipping the technological details that are given in [4-7,11,12].

## Results and discussion

**Figure 1:**
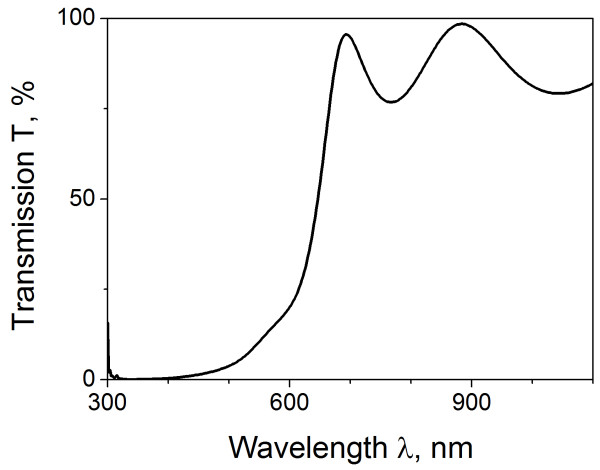
Transmission spectrum of 0.5-μm thick CdSe film.

**Figure 2:**
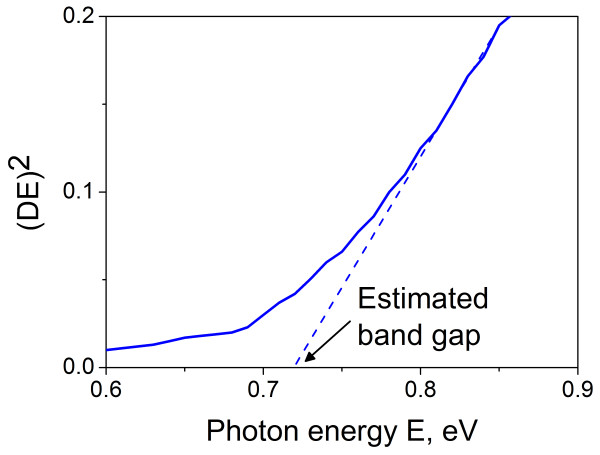
**Diagram used to determine bandgap of PbS CBD sample with growth time of 3 h.** The value of *D* corresponds to optical density.

**Figure 3:**
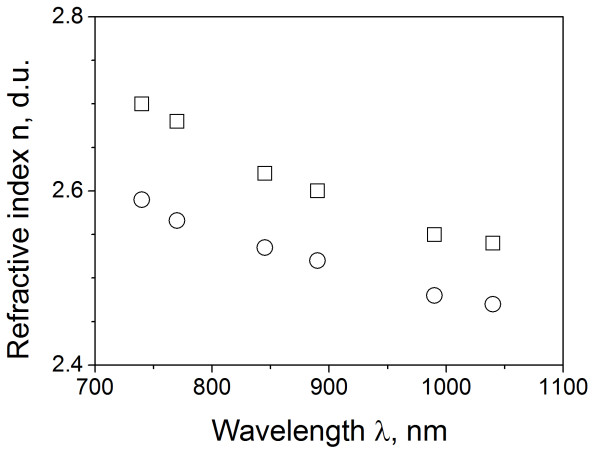
**Refractive index of CdSe.** Squares indicate the data for the bulk material adapted from [14], and circles correspond to CBD film.

**Figure 4:**
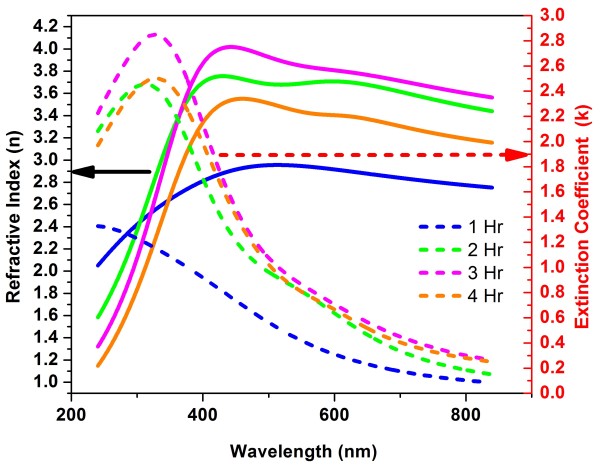
**Optical constants*n, k*of PbS CBD films with different deposition times.**

For CBD-made materials obtained after long deposition time (which resulted into dense films with crystallite size of about 20 nm), we observed a blueshift of the fundamental absorption edge relative to the bulk material data [16] in all cases with the following shift values: 0.06 eV for CdS [7], 0.15 eV for CdSe [6] (see also Figure 1), and 0.1 to 0.4 eV for different samples of PbS (Figure 2). This effect was accompanied by reduction of refractive index *n* (in comparison with bulk crystal data, see Figure 3 for CdSe and Figure 4 for PbS). This reduction is larger for samples obtained with small deposition times, but it is always present in the films discussed here. We connect both effects with pronounced porosity of the films obtained by CBD method. In particular, the blueshift in the dense CBD films is attributed to the quantum confinement in pores. 

Figure 1 presents the transmission spectrum of 0.5-μm-thick CdSe films (deposition time of 4 h) displaying a clear interference pattern, characterized with transmission maxima at 2*dn* = *Nλ* and minima at 2*dn* = (*N −* 1/2) *λ*. Here, *λ* is the wavelength, *d* is the film thickness, and *N* is an integer defining the order of interference pattern. With these expressions, we calculated the spectrum of refractive index (Figure 3, circles). The squares in the same figure present the data for the bulk material [17] displaying a considerable drop of refractive index for the film in comparison with bulk material.

Figure 2 presents the diagram for PbS allowing to determinate the bandgap via direct interband transitions observed for all the materials studied by plotting the squared product of optical density and photon energy as a function of the latter. The similar diagrams for CdS and CdSe were given in [6,7].

**Figure 5:**
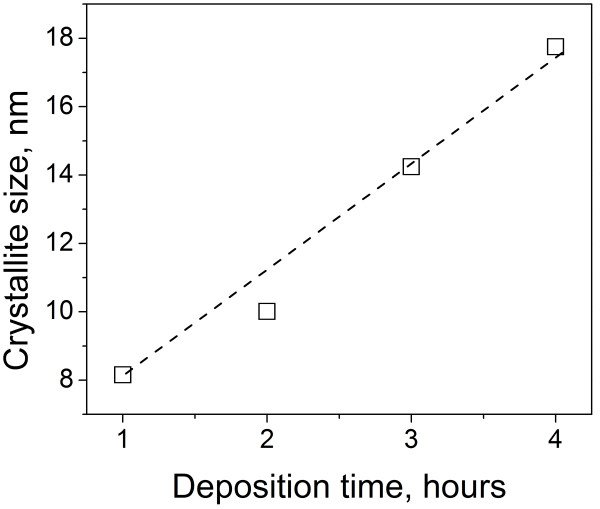
**Dependence of the grain size of PbS CBD samples on growth time.** The line is given as eye guide only.

The case of PbS requires more attention. Figure 5 presents the dependence of the crystallite size upon the deposition time. Figure 4 shows the spectra for optical constants (refractive index *n* and extinction coefficient *k*) measured for four PbS films deposited with growth time ranging from 1 to 4 h; in the latter case, the result was a 100-nm-thick film. It is clear that for larger deposition time, the film becomes denser so that refraction index and extinction coefficients increase. Their spectral behavior follows qualitatively the corresponding curves of the bulk material, but the values are essentially lower, even when deposited film has a considerable thickness. For example, the refractive index for film is 4 at most for the wavelength 450 nm, whereas for the bulk material, the corresponding value is 4.3. As for extinction coefficient *k,* the maximum of 2.75 is achieved at the wavelength of 350 nm, with the corresponding bulk value of 3.37.

We assume that the pores in a dense CBD film correspond to the spaces between crystallites' boundaries. Therefore, in cubic crystals, the pores most probably will be of prismatic shape, defined by plane boundaries of the individual grains. These prismatic pores most probably will have a length (height) equal to the grain size, with quadratic or rectangular triangle cross-section. As pores and crystallites are considered to be of equal height, the question of a volume fraction of pores reduces to two dimensions by being equal to the ratio of pore cross-sectional area to the total cross-section of the film, assuming that in the average there will be one pore per one crystallite. The dimensions of the pore will define the blueshift observed, which can be seen from the following theoretical consideration.

### Electron confined in pores: quantum mechanical approach

It was proposed (see [13-15]) to treat semiconductor quantum dots (QDs) as ‘mirror-wall boxes’ confining the particle, resulting in mirror boundary conditions for analytical solution of the Schrödinger equation in the framework of the effective mass approximation. The basic assumption is that a particle (an electron or a hole) is specularly reflected by a QD boundary, which sets the boundary conditions as equivalence of particle's *Ψ*-function in an arbitrary point r inside the semiconductor (*Ψ*_r_) with wave function in the image point im (*Ψ*_im_). It must be mentioned that *Ψ*-function in real and image points can be equated by its absolute values since the physical meaning is connected with |*Ψ*|^2^, so that mirror boundary conditions can have *even* and *odd* forms (*Ψ*_r_ = *Ψ*_im_ in the former case, and *Ψ*_r_ = −*Ψ*_im_ in the latter). The ‘odd’ case is equivalent to the *impenetrable boundary* conditions and *strong confinement* because *Ψ*-function vanishes at the boundary. The milder case of *even* mirror boundary conditions represents *weak confinement* and occurs when a particle is allowed to have tunnel probability inside the boundary.

It is evident that our basic assumption is favorable for effective mass approximation as it increases the length of effective path for a particle in a semiconductor material. Besides, in high symmetry case, the assumption of mirror boundary conditions forms a periodic structure filling the space. We have shown [15] that the use of even mirror boundary conditions gives the same solution as Born-von Karman boundary conditions applied to a periodic structure. The treatment performed in [13-15] of the QDs with different shapes (rectangular prism, sphere and square base pyramid) yielded the energy spectra that have a good agreement with the published experimental data achievable without any adjustable parameters.

Let us consider an inverted system: a pore formed by a void surrounded by a semiconductor material. The reflection accompanied with a partial tunneling into QD boundary (for the case of *even mirror boundary conditions*) can be described as equivalence of *Ψ*-function values in a real point in the vicinity of the boundary and a reflection point in a mirror boundary. Hence, the solution of the Schrödinger equation for a pore within semiconductor material will be the same as that for a QD of equal geometry with an equal expression for the particle's energy spectrum.

Table 1 summarizes the expressions for energy spectra obtained for QDs of several basic shapes with application of even mirror boundary conditions. All spectra have the same character, with a quadratic dependence on quantum numbers (all integers or odd numbers for a particular case of spherical QD [15]) and an inverse quadratic dependence on QD's dimensions. Besides, the position of energy levels has an inverse dependence on the effective mass [18,19]. 

**Table 1:** Energy spectra of different QDs

**Shape of a QD**	**Cube, side* a ***	**Prism (square base, side* a * ), height * c * > >* a ***	**Sphere, diameter* a ***	**Prism, triangular base (side* a * ), height * c * > >* a ***
Energy spectrum	$E=\frac{3}{8}\frac{h^{2}n^{2}}{ma^{2}}$	$E=\frac{1}{4}\frac{h^{2}n^{2}}{ma^{2}}$	$E=\frac{1}{8}\frac{h^{2}{\left(2n+1\right)}^{2}}{ma^{2}}$	$E=\frac{h^{2}n^{2}}{2ma^{2}}$

Here, *n* is a quantum number, and *m* is the effective mass of the particle.

### Comparison with the experiment

In the following discussion, we take into account that typical pores in CBD materials have a characteristic size *a* of several nanometers [3,9], being much smaller than the Bohr radius *α*_*B*_ for an exciton, *α*/2 < <*α*_*B*_, which is especially important for the case of exciton formation under the action of a light beam incident on semiconductor. The energy difference defines the blueshift of absorption edge. In all semiconductors studied, the value of *α*_*B*_ exceeds 15 nm according to the expression below:

(1) $$a_{B}=\frac{4\;\pi \;\hbar^{2}\;\epsilon \;\epsilon_{0}}{\mu \;e^{2}}with\;reduced\;mass\;\mu =\frac{m_{e}m_{h}}{m_{e}+m_{h}}$$

Here, *m*_e,h_ is the electron/hole effective mass, *ϵ* is the dielectric constant of the material, and *ϵ*_0_ is a permittivity constant. Following the argumentation given in [18,19], we see that one can directly apply the expressions for energy spectra because the separation between the quantum levels proportional to *ħ*^*2*^*/ma*^*2*^ is large compared to the Coulomb interaction between the carriers which is proportional to *e*^2^/*ϵϵ*_0_*α*. Therefore, Coulomb interaction can be neglected, and the energy levels could be found from quantum confinement effect alone. Accordingly, we shall calculate the emission/absorption photon energy for transitions corresponding to the exciton ground state, which is given by *n* = 0 for spherical QD and *n* = 1 for other geometries. From Table 1, it follows that the lowest energy value can be obtained for a spherical QD, whereas for a prism with quadratic section, the energy value is twice larger. For all other geometries, the energy has the latter order of magnitude. For the estimation of porosity effects, we will use the expression for a prismatic QD with a square base, assuming that the fundamental absorption edge corresponds to generation of an exciton with ground state energy:

(2) $$\hbar \omega_{\min}=E_{g}+\frac{h^{2}}{4\mu a^{2}}$$

with the semiconductor bandgap *E*_*g*_.

In the case of CdSe (exciton reduced mass of 0.1 *m*_0_) using the expression (2) and the band edge shift *ħω*_min_ − *E*_*g*_ = 0.15 eV (1.88 − 1.73), we calculate the pore size of 7 nm. For the average crystallite dimension of 22 nm, the pore fraction, thus, would be (7/22)^2^ ≈ 10% , which is twice as big as the relative reduction of refractive index found (Figure 3).

To explain the edge shift observed in CdS (exciton reduced mass 0.134 *m*_0_[16]), one obtains the pore size of 8 nm. Here, the crystallite size is 20.1 nm, making the total pore fraction of approximately 12% . The observed reduction of refractive index changes from 2.5 for the bulk material [7,16] to 2.3 for 600-nm-thick film, yielding the pore fraction of 9% that is close to our predictions.

The reduced mass for PbS is 0.0425 *m*_0_[16], and the observed edge shift is 0.4 eV, yielding the average pore size of 6.5 nm. Having the crystallite size of 20 nm, it will give the pore fraction of 10% (observed reduction of refractive index in [7] was 8% , and from Figure 4 we obtain the value of 7.5% ). We see that in all cases, the volumetric percentage of pores calculated using the blueshift values renders the correct order that is verified from the refractive index reduction. However, the latter value is always smaller that may mean that the pores' height is about 30% to 40% less than that of the grains.

It should be noted that in cases of PbS, due to high value of dielectric constant (17) and small exciton reduced mass, the Bohr radius for an exciton (21 nm) appears to be the same order of magnitude as the grain size. It means that the quantum confinement effect can be observed even without taking into account the porosity of the material. This effect was studied experimentally in [20] for PbS spherical quantum dots. It was found that in PbS quantum dots with diameter of 3.5 nm, the blue band edge shift of 1.05 eV is observed. Taking into account that the blueshift due to quantum confinement is reversely proportional to the square of the dot's diameter, we find that the shift caused by the crystallite size of 20 nm will be equal to 0.03 eV, which is about 10 times smaller than the observed values. We also note that the smaller crystallite size observed in our experiments at early stages of CBD process (variation from 8 to 18 nm, see Figure 5) does not allow to explain the experimentally observed blueshift. Thus, we conclude the mandatory accounting of nanopores, which offers improved agreement between theoretical and experimental data.

## Conclusion

We report on ammonia-free CBD method that provides cheap, efficient, and environmentally harmless production of CdS, CdSe, and PbS films. Material porosity inherent to CBD technique can be used to fine-tune the material bandgap towards the required values, paving promising ways for solar cell applications. The theoretical description of porosity based on the solution of the Schrödinger equation with even mirror boundary conditions provides a good correlation of theoretical and experimental data.

## Competing interests

The authors declare that they have no competing interests.

## Authors' contributions

YVV suggested the treatment of pores as inverted quantum dots. PPH realized the theoretical description and drafted the manuscript. JHB conducted the experiments on CdS and PbSe. HEEP made the experiments on CdSe. RRB adjusted the chemical part of CBD method and helped in drafting the manuscript. PV performed modeling of a porous semiconductor. CP realized the experiments with PbS. JGH supervised all the study. All authors read and approved the final manuscript.
